# Cardiovascular risk estimation using the steno type 1 risk engine in Brazilian individuals with type 1 diabetes: a multicenter cross-sectional study

**DOI:** 10.1016/j.clinsp.2026.101082

**Published:** 2026-09-13

**Authors:** Marília Brito Gomes, Fernanda Oliveira Braga de Albuquerque, Alessandra Matheus, Luiz Henrique Canani, Adriana Costa Forti, Reine Marie Chaves, Elizabeth João Pavin, Renan Montenegro Junior, Sergio A. Dib, João S. Felicio, Rosangela Roginski Réa, Marcia Nery, Carlos Antonio Negrato, Luana Casari Silva

**Affiliations:** a Universidade do Estado do Rio de Janeiro (UERJ), Rio de Janeiro, RJ, Brazil; b Universidade Federal do Rio Grande do Sul (UFRGS), Porto Alegre, RS, Brazil; c Centro de Diabetes de Ceará, Fortaleza, CE, Brazil; d Centro de Diabetes de Salvador, Salvador, BA, Brazil; e Universidade Estadual de Campinas (UNICAMP), Campinas, SP, Brazil; f Universidade Federal do Ceará (UFC), Fortaleza, CE, Brazil; g Universidade Federal de São Paulo (UNIFESP), São Paulo, SP, Brazil; h Universidade Federal do Pará (UFPA), Belém, PA, Brazil; i Universidade Federal do Paraná (UFPR), Curitiba, PR, Brazil; j Universidade de São Paulo (USP), São Paulo, SP, Brazil; k Faculdade de Medicina de Bauru, Universidade de São Paulo, São Paulo, SP, Brazil; l Núcleo de Pesquisa e Ensino IBCC Oncologia, São Paulo, SP, Brazil

**Keywords:** Diabetes type 1, Cardiovascular disease, Steno type 1 risk engine

## Abstract

**Objective:**

To assess the 5- and 10-year Cardiovascular Disease (CVD) risk among Brazilian patients with Type 1 Diabetes (T1D) using the Steno Type 1 Risk Engine (ST1RE), and to evaluate the prevalence of clinical CVD, analyzing traditional and non-traditional risk factors.

**Material and methods:**

This was a cross-sectional multicenter study conducted in 13 public clinics in 11 Brazilian cities, involving 1,760 patients. The ST1RE was used in patients without previous CVD (*n* = 1,300).

**Results:**

The prevalence of CVD was 2.1%, with ischemic heart disease being the most common. These patients were older, had higher age at T1D diagnosis, and longer diabetes duration when CVD was diagnosed. According to ST1RE 10-year risk, 70.7% were classified as low, 18.0% as moderate, and 11.3% as high risk without differences regarding sex. A lower use of cardioprotective drugs was noted in patients with moderate/high 10-year ST1RE in relation to guidelines recommendations. Higher age at diagnosis, presence of retinopathy, elevated high-sensitivity C-reactive protein, and uric acid levels were associated to moderate/high ST1RE risk.

**Conclusions:**

Almost 30% of our patients had moderate/high ST1RE risk and were undertreated for CVD risk factors. This highlights the need to establish strategies to improve cardiovascular and diabetes management risk in routine clinical practice.

## Highlights

• Individuals with type 1 diabetes face high cardiovascular risk and mortality.
• Steno type 1 risk engine helps identify cardiovascular risk in type 1 diabetes.
• Retinopathy, late diagnosis, hs-CRP, and uric acid can be correlated to high cardiovascular risk.
• Cardioprotective drugs are underused in moderate and high-risk Brazilian individuals with T1D.
• Identifying risk factors may improve prevention of cardiovascular disease in individuals with T1D.

## Introduction

Type 1 Diabetes (T1D) is recognized as a chronic autoimmune disease with an increasing incidence worldwide. ^1^ In 2021, an estimated 8.1 million individuals received a diagnosis of T1D, with most cases identified during childhood and a significant number in adulthood.^2^ As it is a lifelong disease, patients present an increased risk of morbidity due to chronic-related complications^3^^,^^4^ and high mortality rates mainly due to Cardiovascular Disease (CVD).^5^ Although an improvement in life expectancy for patients with T1D has been observed in the last decades,^6^ these patients still have an excess mortality of up to 5-fold compared with the general population.^6^ These high mortality rates lead to a 12-year decrease in life expectancy,^6^ associated with Cardiovascular Risk Factors (CVRF) in different degrees.^6^

The prevalence of CVD in patients with T1D was about 10% in the EURODIAB study, with a noted variation across European centers.^7^ In this study, CVD was associated with higher age, longer diabetes duration, and the presence of traditional CVRF, such as hyperglycemia, hypertension, dyslipidemia, and non-traditional CVRF, such as obesity, insulin resistance, and chronic inflammation.^7^

Considering several studies performed in different countries as Europe,^7^ USA,^8^ and Brazil,^9^ most patients with T1D did not meet the goals for blood pressure and LDL-cholesterol, and even glycemic control. Blood pressure, LDL-cholesterol, and glycemic control are CVRF,^7^ pointing out CVD prevention as a challenge in routine clinical practice.^8^

All the data above provide relevant information about the need to stratify patients with T1D for CVD risk, which could be used for prognostic and therapeutic interventions.^10^ Although several prediction risk calculators have been developed for patients with type 2 diabetes, such tools did not perform adequately for patients with T1D^11^ due to missing variables in the prediction equation that are relevant for these patients, such as diabetes duration, age at diagnosis, and glycemic control.

The most used calculators for the prediction of CVD risk in patients with T1D are the Steno Type 1 Risk Engine (ST1RE),^12^ the Sweden National Register,^13^ the European Society of Cardiology (ESC),^14^ the QRISK3 (ClinRisk, UK),^15^ a calculator developed in Scotland^16^ and another developed in Brazil.^17^ In general, comparative studies using different risk calculators showed low agreement primarily due to different cut-off values for age and diabetes duration and differences in the variables used in the prediction equations.^18^ The ST1RE has already been used and validated in patients with T1D from Brazil,^19^ Italy^20^ and Denmark.^21^ It has also been used in Spain as an additional tool for diagnosing preclinical atherosclerosis.^22^

The primary objective of this multicenter observational study carried out with Brazilian patients with T1D was to determine the estimated 5- and 10-year CVD risk using the ST1RE, and the secondary goal was to evaluate the prevalence of clinical CVD. For both objectives, we aimed to establish the association with traditional and non-traditional CVRF.

### Study design, participants, and methods

This was a retrospective, descriptive, cross-sectional, multicenter study conducted between August 2011/August 2014 in 13 public clinics that participated in the Brazilian Type 1 Diabetes Study Group (BrazDiab1SG) in 11 cities from all Brazilian geographic regions. Research design and methods have been previously detailed.^23^ Briefly, each clinic provided data from at least 50 outpatients with T1D receiving healthcare from the Brazilian National Health Care System (BNHCS). The sample included 1,760 patients diagnosed with T1D between 1960 and 2014, as described in Supplementary Table 1. This study was approved by the local ethics committee of each center, and written informed consent was obtained from all patients and/or their parents where necessary. The patients diagnosed with T1D according to the American Diabetes Association (ADA) criteria^24^ were included. Pregnant or lactating women, patients with an acute infection or ketoacidosis in the three preceding months before recruitment were not eligible for the original study. Patients younger than 18-years old were excluded from the current analysis.

Participants completed a standardized questionnaire during a clinical visit, which evaluated clinical and demographic data such as sex, current age, birthplace, self-reported race/skin color (Black, White, Brown, Yellow, and Indigenous),^25^ age at diagnosis, diabetes duration, years of school attendance, current smoking status (at least ≥ one cigarette per day), self-reported frequency of physical exercise (at least 3,5 hours/week), BMI (determined by dividing an individual’s weight [kg] by the square of the height [m^2^]). Brachial systolic and diastolic blood pressure was measured twice in a sitting position by an automated oscillometric device (OMRON® HEM 742INT). An optimal blood pressure was defined as < 130/80 mmHg.^14^^,^^24^

The type of prescribed Insulin Therapeutic Regimens (ITR) was stratified as follows: exclusive use of intermediate insulin (NPH) or long-acting insulin analogs, use of intermediate insulin (NPH) plus regular insulin or short-acting insulin, use of long-acting insulin analogs plus short-acting insulin, or use of Continuous Subcutaneous Insulin Infusion (CSII), use of lipid-lowering or antihypertensive drugs.

Hypertension was defined as sustained blood pressure ≥ 140/90 mmHg or the current use of antihypertensive drugs.^9^ The diagnosis of Metabolic Syndrome (MS) was made according to the International Diabetes Federation criteria.^26^

A1C, creatinine, urea, triglycerides, total cholesterol, HDL cholesterol, LDL cholesterol, and uric acid were measured using enzymatic techniques (BioSystem, model A25; Barcelona, Spain). A1C was measured using High-Performance Liquid Chromatography (HPLC, Bio-Rad Laboratories, Hercules, California, USA). Friedewald’s equation was used to calculate Low-Density Lipoprotein cholesterol (LDL cholesterol) values.^27^ The total cholesterol/HDL-cholesterol ratio was calculated. Creatinine was measured using a colorimetric assay kit, corrected for a standardized creatinine assay by mass spectrometry, and C-Reactive Protein (hs-CRP) was measured by immunoturbidimetry, a high sensitivity enzyme immunoassay (Biosystems). All the above laboratory data were measured in a single center (State University of Rio de Janeiro).

### Evaluation of renal function and retinopathy

Renal function was estimated by the CKD-EPI equation^28^ in adults and was expressed as estimated Glomerular Filtration Rate (eGFR) in milliliters per minute per 1.73 m^2^ (mL/min). We considered all patients as non-African Americans in the CKD-EPI equation. Albuminuria concentration was measured from a morning urine sample by immunoturbidimetry. The presence of albuminuria was defined as albuminuria ≥ 30 mg/dL, and patients were stratified into three groups: normoalbuminuric, microalbuminuric (30‒299 mg/dL) and macroalbuminuric (≥ 300 mg/dL).^24^

The screening for Diabetic Retinopathy (DR) was performed by mydriatic Binocular Indirect Ophthalmoscopy (BIO). The classification of DR for each patient was assessed for the eye with the highest level of commitment. Each eye was classified based on the absence or presence of DR, which was classified as absent, Non-Proliferative DR (NPDR), Proliferative DR (PDR), and macular edema, according to the international classification of DR.^29^ Afterward, DR was stratified as referable (moderate non-proliferative DR, severe non-proliferative DR, proliferative DR, and macular edema) and non-referable (DR absent and mild DR).^29^

The risk of presenting fatty liver was determined by the Fatty Liver Index (FLI).^30^ Participants with FLI ≥ 60 and < 30 were classified as having high and low risk of having a fatty liver, respectively. Values between 30 and 60 were considered as undetermined risk.^30^

### Cardiovascular disease (CVD)

Data regarding clinical CVD were retrieved from medical records and defined as follows: ischemic heart disease (ICD-10-I20-I25), including angina (ICD-10-I20), ischemic stroke (ICD-10-I64), heart failure (ICD-10-I50), and peripheral artery disease (ICD-10-I73.9).

### Prediction of cardiovascular risk

Prediction of cardiovascular risk was performed according to the ST1RE, which includes ten risk factors: age, sex, diabetes duration, systolic blood pressure, LDL-cholesterol, A1C, albuminuria, eGFR, smoking, and exercise practice. For this analysis, eligible patients were those adults without clinical CVD^12^ and without any missing data for all the ten variables above described.

The overall ST1RE results for the 5-years were performed and the ST1RE results for 10-year CVD risk were then categorized as low (< 10%), moderate (10%‒20%), and high (≥ 20%).^12^ The moderate and high-cardiovascular risk patients were then grouped to analyze adherence to blood pressure and LDL-cholesterol goals. The 2014 ADA standards of care were used to determine the Blood Pressure (BP less than 140/80 mmHg) and lipid (LDL cholesterol less than 100) targets as this was the guideline used at the end of data collection.^24^

### Identification of factors associated with moderate/high ST1RE risk

A prediction model was created to identify variables that could be associated with moderate/high cardiovascular risk according to the ST1RE engine. The outcome was dichotomized between moderate/high and low cardiovascular risk. The potential predictors included in the model were the following: age at diagnosis as a binary variable (< 19 vs. > 19-years of age), BMI as a binary variable (≥ 25 vs. < 25 Kg/m^2^), educational level as a binary variable (years of school attendance > 12 vs. ≤ 12-years), presence of fatty liver as a categorical variable (FLI < 30, FLI ≥ 30 & < 60, FLI ≥ 60), triglycerides as a binary variable (< 150 vs. ≥ 150 mg/dL), uric acid as a binary variable (< 5.2 vs. ≥ 5.2 mg/dL), presence of DR as a binary variable and also hs-CRP as a binary variable (< 0.2 vs. ≥ 0.2 mg/dL). These variables were selected based on previous studies demonstrating their association with an increased cardiovascular risk in patients with T1D.^26^^,^^29^, ^30^, ^31^, ^32^, ^33^, ^34^ The chosen cut-offs were derived from scientific literature that identified diabetes onset during childhood (< 19-years of age),^27^ overweight status (BMI ≥ 25 kg/m^2^),^26^ and triglycerides of 150 mg/dL or above^26^ as risk factors for cardiovascular disease in individuals with T1D. The cutoff point of 0.2 mg/dL for hs-CRP is based on population data showing an increased cardiovascular risk starting above this threshold.^35^ FLI was categorized to reflect low, intermediate, and high risk of fatty liver disease.^30^ For serum uric acid, 5.2 mg/dL and above was chosen as a cutoff based on a previous study that showed an association between these levels and a decline in renal function in Brazilian T1D patients.^32^ Educational level was dichotomized at 12-years, corresponding to college admission, according to the Brazilian educational system.^36^

### Sample calculation, economic status

A detailed description of the study sample calculation has been described previously.^23^ Briefly, the study sample represented the distribution of T1D cases across four geographic regions of Brazil. This distribution was estimated using the overall population distribution reported in the 2000 Brazilian Institute of Geography and Statistics Population Census (IBGE).^25^ These data were combined with national estimates of diabetes prevalence derived from a 1988 survey to determine the minimum number of patients to be studied in each region.^37^ Economic status was defined according to the Brazilian Economic Classification Criteria.^38^ The following economic status categories were considered for this analysis: high, middle, low, and very low.

### Statistical analysis

A descriptive analysis assessed clinical, demographic, and laboratory data stratified according to 10-year ST1RE (low, moderate, and high risk). These data were presented as means (± SD) or median, Interquartile Range [IQR] or minimum and maximum for continuous variables, and as counts (relative frequencies) for discrete variables. For categorical variables analysis, the Chi-Square test was used. We used Anova with Sidak correction for multiple comparisons and Student’s *t*-test for continuous variables.

A stepwise regression algorithm with backward selection was used to identify predictors associated with moderate/cardiovascular risk according to ST1RE. A p-value of 0.01 was used to adjust for multiple comparisons. A fit logistic regression model containing all potential predictors was created. The least significant predictor was eliminated, and a new model with the remaining variables was fitted. This process was repeated until the final model had only predictors with p-values under 0.01.

Apart from the prediction rule to identify predictors of moderate/high cardiovascular risk individuals according to ST1RE, all analyses were performed using the Statistical Package for the Social Sciences (SPSS version 17.0, SPSS, Inc., Chicago, Illinois, USA). A two-sided p*-*value of less than 0.05 was considered significant, whereas a two-sided p-value of less than 0.01 was used to adjust for multiple comparisons. STATA/BE software was used to create prediction equations.

## Results

### Overview of the individuals with clinical CVD

**Fig. 1:**
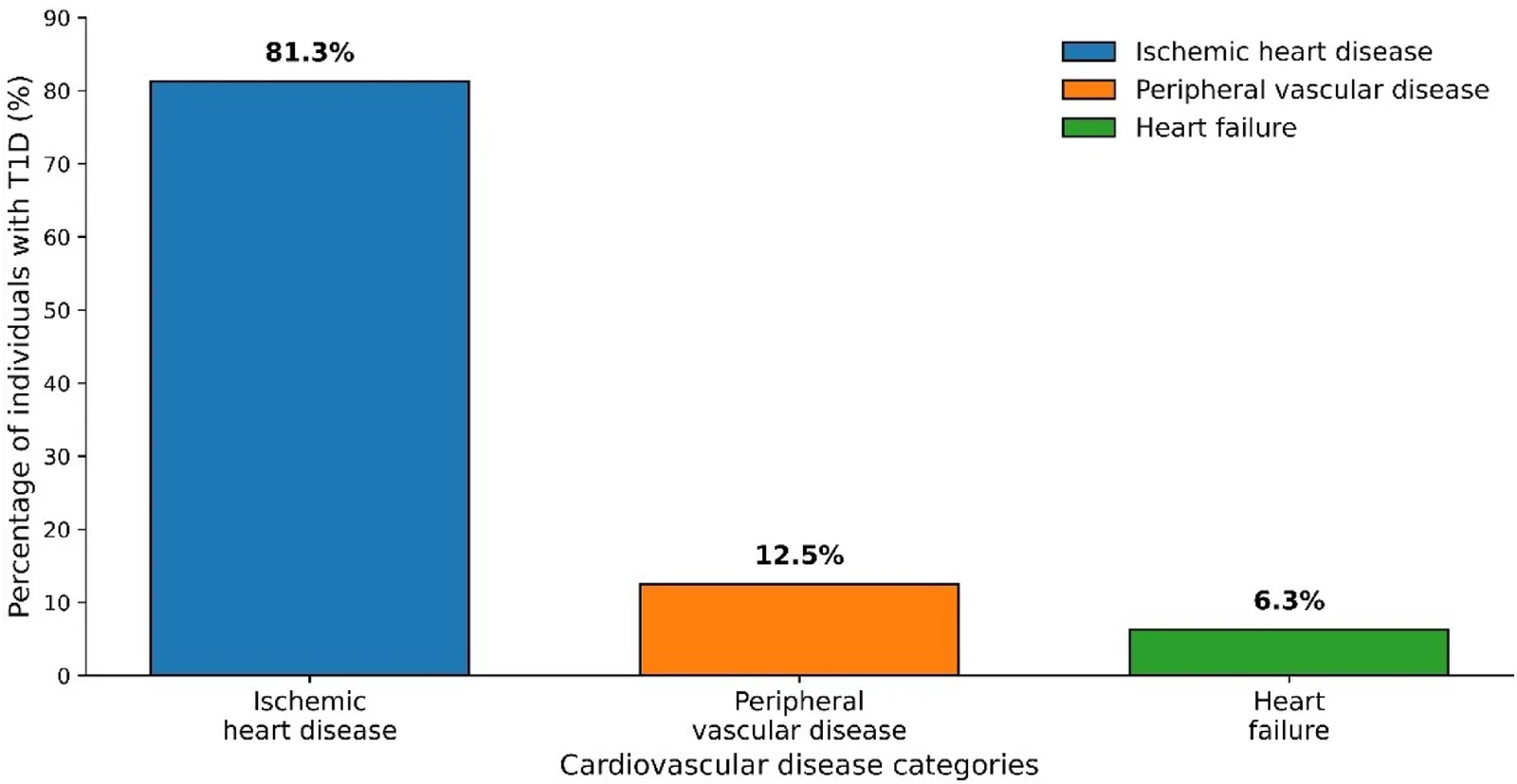
Distribution of individuals with T1D (%) according to the type of cardiovascular disease.

The sample of this study was comprised of 1,521 individuals after the exclusion of adolescents (*n* = 239). Overall, 32 patients (2.1%) were diagnosed as having CVD, mainly Ischemic Heart Disease (IHD), n = 26 (81.3%). Peripheral vascular disease was observed in four patients (12.5%) and heart failure in two (6.3%) (Fig. 1). Compared to patients without CVD, these patients were older, had higher age at diabetes diagnosis, and longer diabetes duration (Table 1). Most of them were male [(*n* = 19 (59.4%)], older than 40-years [(*n* = 24 (75%)] and had a diabetes duration at CVD diagnosis between 15 to < 30-years [n = 25 (78.1%)]. Moreover, they had longer follow-ups in their respective diabetes centers and presented larger waist circumference, higher BMI, SBP, FLI, and lower eGFR. Hypertension and DR were more common in these patients, as well as referable DR. Regarding drug treatments, these patients were more commonly receiving statins, antihypertensive drugs, and metformin. No difference was noted in A1C levels.

**Table 1:** Clinical, demographic and laboratory data stratified by the presence of clinical macrovascular disease.

	Cardiovascular disease		
	No	Yes	ap-value
n (%)	1489 (97.9)	32 (2.1)	
**Demographic data**			
Gender, male n (%)	625(42.0)	19 (59.4)	0.049
Age, y	31.9 ± 10.9	49.2 ± 9.9	<0.001
Diabetes duration, y	16.5 ± 8.9	30.5 ± 10.8	<0.001
Diabetes duration at CVD, y		24.3 ± 9.2	
Age at diabetes diagnosis, y	16.5±8.9	19.2 ± 7.6	0.01
Age at CVD, y	‒	43.7±8.2	
Time of follow up, y	8.3 [9.3]	17.5 [17.4]	0.002
Level of care, tertiary n (%)	957 (64.3)	22 (68.8)	0.4
Health care insurance (public and private), yes n (%)	459 (30.8)	10 (31.3)	0.05
Years of study, y	12.5 ± 8.9	12.2 ± 3.5	0.5
Smoker, yes n (%)	77 (5.2)	4 (12.5)	0.07
**Ethnicity, y (%)**^b^			
Caucasian	832 (55.9)	18 (56.3)	0.9
**Economic Status (%)**			0.5
High	45 (3.0)	1 (3.1)	
Medium	704 (47.3)	13 (40.6)	
Low	696 (46.7)	17 (53.1)	
Very Low	44 (3.0)	1 (3.1)	
**Diabetes management and treatment**			
HbA1c (%)	9.01 ± 2.2	8.5 ± 1.9	0.2
HbA1c (mmoL/moL)	73.6 ± 22.3	69.0 ± 20.9	
Insulin dose (U/kg/day)	0.83 ± 0.4	0.80 ± 0.31	0.6
SMBG, yes n (%)	1400 (94.0)	31 (96.9)	0.3
SMBG, n	3.6 ± 1.4	3.4 ± 1.2	0.3
Adherence to diet, yes n (%)	783 (59.9)	20 (66.7)	0.4
Physical activity, yes n (%) c	739 (49.7)	13 (40.6)	0.3
Number of clinical visits/year	3.6 ± 1.7	3.9 ± 1.9	0.3
**Clinical data**			
BMI, kg/m^2^	24.6 ± 4.1	26.2 ± 4.9	0.03
Waist circumference (WC), cm	83.7 ± 11.4	88.8 ± 11.7	0.01
Overweight/obesity, yes n (%)	583 (39.2)	15 (46.9)	0.38
Systolic blood pressure	123.1 ± 15.8	130.7 ± 22.7	<0.001
Diastolic blood pressure	76.2 ± 10.9	77.2 ±11.3	0.6
Hypertension, yes n (%)	290 (19.5)	21 (65.6)	<0.001
Metabolic syndrome, yes n (%) f	485 (33.3)	16 (50.0)	0.048
Fatty liver index	16.2 [28.1]	35.9 [52.9]	0.017
**Laboratory data**			
Uric acid (mg/dL)	5.1 ± 1.9	5.7 ± 1.7	0.058
Total Cholesterol (mg/dL)	188.8 ± 49.4	173.9 ± 72.2	0.26
LDL-cholesterol (mg/dL)	110.4 ± 40.2	100.7 ± 47.7	0.17
LDL-cholesterol < 100 mg/dL), yes n (%)	645 (44.4)	17 (54.8)	0.24
Triglycerides (mg/dL)	86 [62.0]	93 [55.0]	0.5
HDL-cholesterol (mg/dL)	56.8 ± 19.2	56.6 ± 23.9	0.9
Ratio Total Cholesterol/HDL-cholesterol	3.6 ± 1.4	3.2± 1.04	0.07
Creatinine (mg/dL)	1.03 [0.35]	1.14 [0.41]	0.13
C reactive protein (mg/dL)	0.19 [0.47]	0.15 [0.47]	0.34
**Medications**			
Metformin, yes n (%)	183 (12.5)	11 (34.4)	<0.001
Anti-hypertensive drugs, yes n (%)	436 (29.5)	25 (78.1)	<0.001
Statin yes n (%)	350 (23.5)	27 (84.4)	<0.001
**Diabetes-related chronic complications**			
Retinopathy, yes n (%)	577 (39.9)	28 (87.5)	<0.001
Referrable retinopathy, yes n (%)	273 (19.7)	20 (64.5)	<0.001
GFR, mL/min/1.73m^2^^e^	83.0 ± 27.6	62.1 ± 21.1	<0.001
Albuminuria, mg/dL	9.2 [22.8]	9.4 [22.7]	0.4

The data are presented as n (%), mean ± SD or median [IQR, Interquartile Range]

a p < 0.05 was considered significant; CVD, Cardiovascular Disease

b African Brazilians, Mulattos, Asians, Native Amerindians were considered as non-Caucasians

c Physical activity, at least 3/times per week ^d^FLI: fatty liver index

e Glomerular filtration rate

f Metabolic syndrome was defined according to International Diabetes Federation (WC ≥ 90 cm in South American men or ≥ 80 cm in South American women; triglycerides ≥ 150 mg/dL (1.7 mmoL/L) or on drug therapy for elevated triglycerides; HDL < 40 mg/dL (1.03 mmoL/L) in men or < 50 mg/dL (1.29 mmoL/L) in women or on drug therapy for low HDL; elevated blood pressure (systolic or diastolic) ≥ 130/85 mmHg or the use of antihypertensive drugs).

### Overview of the patients stratified according to ST1RE

**Fig. 2:**
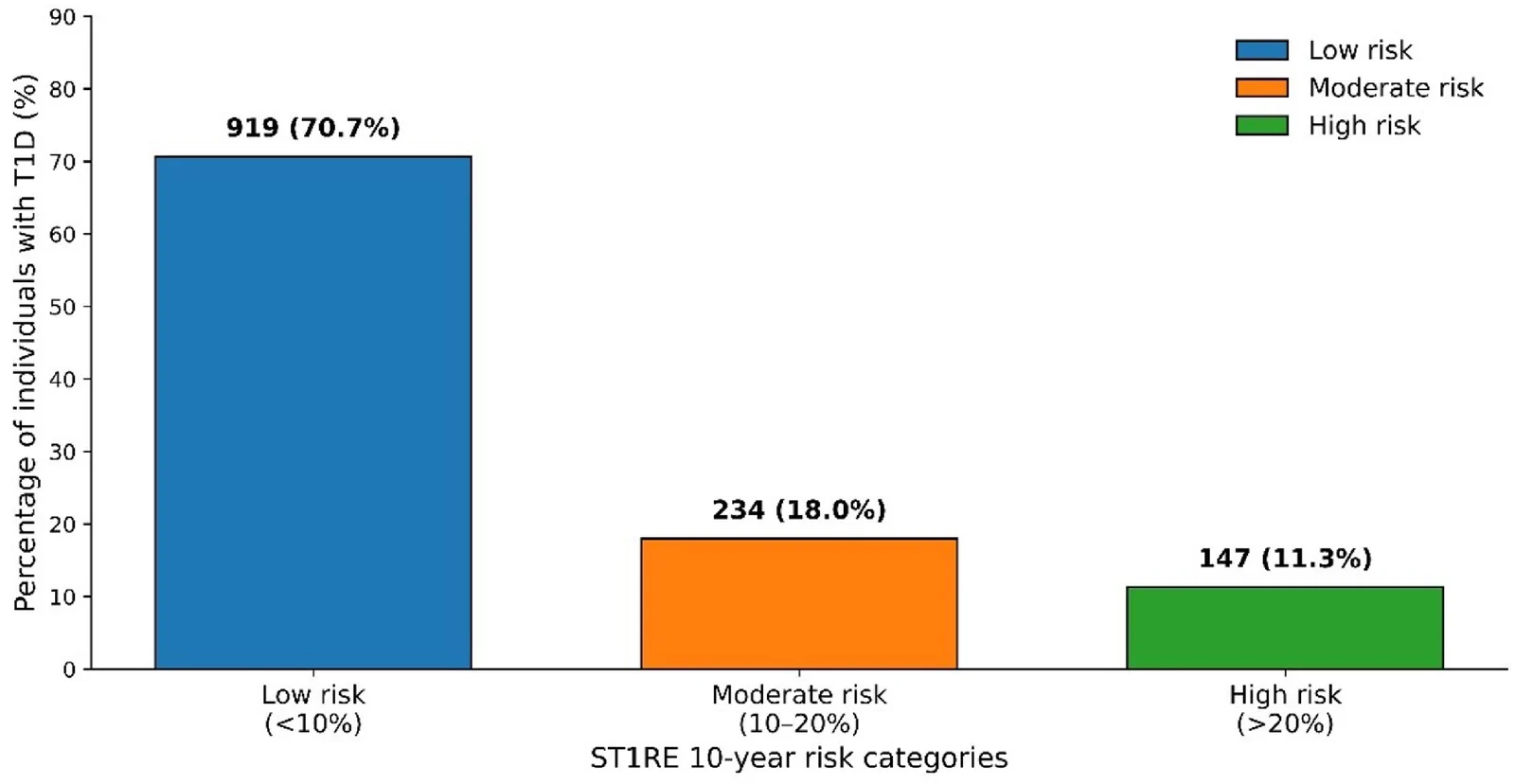
Distribution of individuals with T1D (%) across 10-year cardiovascular risk categories according to the ST1RE.

For this analysis, after the exclusion of adolescents (*n* = 239), exclusion of patients with previous CVD (*n* = 32) and exclusion of the patients with missing data according to the criteria defined by ST1RE^12^ (*n* = 189), 1,300 patients were found to be eligible. The overall median of 5-year and 10-year risk for CVD was 2.7% (4.41) and 5.44% (8.47), respectively. A 10-year moderate and high risk was noted in 234 (18.0%) and 147 (11.3%) patients, respectively (Fig. 2). The comparisons among low, moderate, and high 10-year risk groups are described in Table 2. For those variables that comprised the ST1RE, only sex was not significant. Among the other variables, ethnicity, level of care, LDL-cholesterol levels within the target, cholesterol/HDL-cholesterol ratio, and use of metformin did not reach statistically significant results. A significant difference was observed in the frequency of FLI ≥ 60 among low, moderate, and high 10-year risk, 80 patients (9.2%) vs. 32 patients (14.3%) vs. 29 patients (20.1%) respectively; p < 0.001. The comparisons between 10-year low and high-risk groups were significant for all the variables described in Table 1.

**Table 2:** Clinical, demographic and laboratory data stratified by 10-year Steno Risk Engine (ST1RE).

		Steno Risk Engines			
All subjects		Low	Moderate	High	ap-value
n (%)	n = 1300	919 (70.7)	234 (18.0)	147 (11.3)	
**Variables used in ST1RE**					
Age, y	32.2 ± 11.0	27.2 ± 6.8	41.9 ± 8.7	47.9 ± 10.5	<0.001
Diabetes duration, y	16.7 ± 8.9	13.9 ± 6.8	21.1 ± 8.9	26.5 ± 10.6	<0.001
Smoker, yes n (%)	70 (5.4)	33 (3.6)	20 (8.5)	17 (11.6)	<0.001
Exercise, yes, n (%) ^b^	644 (49.5)	493 (53.6)	92 (39.3)	59 (40.1)	<0.001
HbA1c (%)	8.9 ± 2.0	8.6 ± 1.9	9.0 ± 2.2	9.5 ± 2.2	<0.001
HbA1c (mmoL/moL)	73.3 ± 22.0	71.5 ± 20.9	75.6 ± 23.9	81.3 ± 23.9	
Systolic blood pressure	123.2 ± 15.6	119.4 ± 12.7	129.4 ± 16.7.7	136.9 ± 19.1	<0.001
LDL-cholesterol (mg/dL)	110.1 ± 39.1	108.7 ± 37.0	111.3 ± 40.4	121.3 ± 47.6	0.001
eGFR, mL/min/1.73m^2^^e^	83.5 ± 27.2	91.3 ± 23.5	71.3 ± 21.5	54.0 ± 24.2	<0.001
Albuminuria, mg/mL	8.7 [20.7]	8.1 [14.9]	9.2 [442.6]	33.3 [123.9]	<0.001
**Other demographic variables**					
Age at diabetes diagnosis, y	15.6 ± 9.2	13.4 ± 7.4	20.6 ± 10.8	21.4 ± 11.1	<0.001
Level of care, tertiary n (%)	869 (66.8)	589 (64.1)	170 (72.6)	110 (74.8)	0.4
Health care insurance (public and private), yes n (%)	401 (30.8)	309 (33.6)	67 (28.6)	25 (17.0)	<0.001
Years of study, y	12.9 ± 3.4	12.9 ± 3.4	11.5 ± 4.8	11.1 ± 4.1	<0.001
Ethnicity, y (%) c					
Caucasian	748 (57.5)	521 (56.7)	134 (57.3)	93 (63.3)	0.18
Economic Status (%)					0.03
High	39 (3.0)	32 (3.5)	7 (3.0)	0	
Medium	631 (48.5)	454 (49.4)	106 (45.3)	71 (48.3)	
Low	596 (45.8)	414 (45.2)	109 (46.6)	72 (49.0)	
Very Low	34 (2.6)	18 (2.0)	12 (5.1)	4 (2.7)	
**Diabetes management and treatment**					
HbA1c < 7.0% n (%)	202 (15.5)	161 (17.5)	32 (13.7)	9 (6.1)	0.4
HbA1c (%) year before	8.8 ± 2.1	8.6 ± 2.0	8.9 ± 2.1	9.4 ± 2.2	0.001
HbA1c (mmoL/moL), year before	72.5 ± 22.7	71.1 ± 22.0	73.9 ± 23.5	78.9 ± 24.6	
SMBG, n	3.7 ± 1.4	3.7 ± 1.4	3.7 ± 1.5	3.6 ± 1.4	0.6
Adherence to diet, yes n (%) d	688 (61.0)	499 (61.0)	108 (54.0)	81 (65.3)	0.9
Number of clinical visits/years	3.7 ± 1.7	3.6 ± 1.7	3.7 ± 1.7	4.0 ± 1.8	0.036
**Other Clinical data**					
BMI, kg/m^2^	24.6 ± 4.1	24.3 ± 3.9	25.3 ± 4.5	25.3 ± 4.2	<0.001
Overweight/obesity, yes n (%)	519 (39.9)	338 (36.8)	119 (49.1)	62 (42.2)	0.008
Diastolic blood pressure	76.2 ± 10.8	74.8 ± 9.8	79.0 ± 12.1	80.7 ± 12.5	<0.001
Hypertension, yes n (%)	257 (19.8)	80 (8.7)	91 (38.5)	86 (58.5)	<0.001
Blood pressure < 140/80mmHg	748 (57.5)	599 (80.1)	121 (51.7)	51 (34.7)	<0.001
Metabolic syndrome, yes n (%)^f^	430 (33.1)	245 (27.2)	106 (46.1)	79 (54.1)	<0.001
Fatty liver index	16.4 [28.5]	14.3 [23.30]	21.4 [32.0]	29.0 [40.4]	<0.001
**Laboratory data**					
Uric acid (mg/dL)	5.1 ± 1.9	4.8 ± 1.6	5.3 ± 2.3	6.2 ± 2.4	<0.001
	188.8 ± 49.3	184.8 ± 44.9	193.8 ± 49.9	209.1 ± 66.5	<0.001
HDL- cholesterol (mg/dL)	56.7 ± 18.7	55.9 ± 17.6	57.9 ± 17.7	59.6 ± 24.5	0.043
Ratio Cholesterol/HDL	3.6 ± 1.4	3.5 ± 0.3	3.6 ± 1.4	3.8 ± 1.9	0.03
LDL-cholesterol < 100 mg/dL) yes n (%)	592 (45.5)	429 (46.5)	108 (46.2)	55 (37.4)	0.068
Triglycerides (mg/dL)	87 [62.5]	81 [57.0]	94 [58.5]	113 [84.0]	<0.001
ALT, U/L	12.0 [9.0]	11.0 [8.0]	12.0 [10.0]	13 [8.0]	0.002
AST, U/L	18.0 [11.0]	17.0 [11.0]	19.5 [9.25]	21.0 [12.0]	0.001
GGT, mg/dL	18.0 [15.0]	18.0 [13.0]	18.0 [15.0]	25.0 [25.0]	0.001
Creatinine (mg/dL)	1.03 [0.33]	0.99 [0.31]	1.07 [0.36]	1.29 [0.73]	<0.001
C reactive protein (mg/dL)	0.2 [0.46]	0.18 [0.44]	0.22 [0.46]	0.28 [0.59]	0.008
**Medications**					
Metformin, yes n (%)	160 (12.3)	106 (11.5)	34 (14.4)	20 (14.4)	0.6
Anti-hypertensive drugs, yes n (%)	391 (30.1)	173 (18.8)	127 (54.3)	91 (61.9)	<0.001
Statin yes n (%)	319 (24.5)	145 (15.8)	96 (41.0)	78 (53.1)	<0.001
Antiplatelet, yes n (%)	138 (10.6)	33 (3.6)	54 (23.1)	51 (34.7)	<0.001
**Other Diabetes-related chronic complications**					
Retinopathy, yes n (%)	502 (38.6)	269 (30.1)	128 (56.1)	105 (72.4)	<0.001
Referrable retinopathy, yes n (%)	241 (18.5)	103 (11.5)	78 (34.2)	60 (41.4)	<0.001

The data are presented as n (%), mean ± SD or median [IQR, Interquartile Range]

a p < 0.05 was considered significant for comparison among low, moderate and high 10- year risk. CVD, Cardiovascular Disease; ALT, Alanine Aminotransferase; AST, Aspartate Aminotransferase; GGT, Gamma-Glutamyl Transferase; FLI, Fatty Liver Index; SMBG, Self-Monitoring of Blood Glucose; BMI, Body Mass Index. ^b^ Exercise, at least 3,5h/week

c African Brazilians, Mulattos, Asians, Native Amerindians were considered as non-Caucasians

d Diet adherence was defined as following at least 80% of the time of the reported diet

e Glomerular filtration rate

f Metabolic syndrome was defined according to International Diabetes Federation (WC ≥ 90 cm in South American men or ≥ 80 cm in South American women; triglycerides ≥ 150 mg/dL (1.7 mmoL/L) or on drug therapy for elevated triglycerides; HDL < 40 mg/dL (1.03 mmoL/L) in men or < 50 mg/dL (1.29 mmoL/L) in women or on drug therapy for low HDL; elevated blood pressure (systolic or diastolic) ≥ 130/85 mmHg or the use of antihypertensive drugs).

Smoking, regular exercise, years of school attendance, BMI, diastolic blood pressure, triglycerides, ALT, AST, and use of antihypertensive drugs or statins were not significantly different between moderate and high 10-year-risk. Moreover, when comparing low and moderate 10-year-risk, LDL cholesterol levels, A1C in the previous year, type of health care, and use of antihypertensive drugs or statins were not statistically different.

Grouping moderate and high cardiovascular risk groups (*n* = 381), 209 individuals presented with blood pressure ≥ 140/80 mmHg. Considering patients with BP out of the target, 37.3% (*n* = 78) were not receiving lowering blood pressure drugs. Moreover, 218 patients in this aggregate group had LDL cholesterol equal to or greater than 100 mg/dL, and among the patients with LDL cholesterol out of the target range, 58.3% were not on statins.

### Identification of other factors associated with moderate/high CV risk in the ST1RE engine

Age at T1D diagnosis over 19-years (OR = 6.3, 95% CI [4.66‒8.51]), hs-CRP^2^ 0.2 mg/dL (OR = 1.46, 95% CI [1.1‒1.94]), presence of DR (OR = 4.25, 95% CI [3.18‒5.68]), and uric acid^2^ 5.2 mg/dL (OR = 1.58, 95% CI [1.18‒2.1]) were associated with moderate/high cardiovascular risk according to St1RE in the final logistic regression model.

## Discussion

In the current study, most patients had low risk in the 5- and 10-year risk ST1RE scores. There was no difference between low, moderate, and high 10-year risk for sex, ethnicity, and LDL-cholesterol within the target. A linear trend was observed for all the other clinical and laboratory variables, including the use of medications and the prevalence of DR across all risk groups (low, moderate, and high risk). Higher age at diabetes diagnosis, presence of DR, and higher levels of hs-CRP and uric acid were associated with 10-year moderate/high risk according to ST1RE.

Moreover, CVD occurred in 2.1% of patients, and the main CVD found was IHD. Most of them had been diagnosed with CVD with an age greater than 40-years (75%) and a diabetes duration between 15 to < 30-years (78.1%). Compared to patients without CVD, these patients were diagnosed with T1D at a higher age, had more time for follow-ups in their respective diabetes centers, larger waist circumference, higher BMI, SBP, and a higher prevalence of the following variables: hypertension, any type of DR and referable retinopathy, use of statins, antihypertensive drugs, and metformin. In addition, they also had a lower eGFR than patients without CVD, and, like EURODIAB, no difference in A1C levels was noted.^7^

Considering the ADA 2014 standards of medical care in diabetes, a significant number of patients in the moderate/high cardiovascular risk group with blood pressure or LDL-cholesterol out of target were not receiving antihypertensive medications or statins, highlighting the sub-optimal treatment of CVRF, including the management of glycemic control in these latter patients.^9^ Studies performed in other countries also showed a gap between clinical guidelines and clinical practice for CV risk control in T1D.^39^ It is important to note, that in the early 2010s, statins were not dispensed in public primary care settings in Brazil. It is important to point out that our study was not designed to identify the putative causes of undertreatment of the above comorbidities. Further studies, approaching also clinical inertia must be conducted in Brazil to clarify this topic.

An overall 5-year risk of 2.76% and 10-year risk of 5.44% in ST1RE^12^ was observed, which was lower than the one described in Italy for 5-year, i.e., 5.9%^20^ and 10-year, i.e.,15.42%.^20^ Moreover, we also noted a percentage of patients classified as 10-year high risk in ST1RE (11.3%) that was lower than that described both in Italy (25.3%)^20^ and Spain (28.4%).^22^ This was primarily due to differences in the eligibility criteria of the studies mentioned above, related to age (> 40y), diabetes duration (> 10y), and the presence of any CVRF compared to our study.

In the present study, we evaluated a general population of patients with T1D. As expected, as the estimated 10-year risk increased, we observed a worsening of concentrations and proportion values of the variables included in ST1RE, except for sex. As the 10-year risk increased, many clinical and laboratory variables not included in the ST1RE showed a worsening in their values. Moreover, the higher the 10-year risk, the higher the prevalence of comorbidities, such as hypertension, and diabetes-related chronic complications, such as DR. However, the increased use of medications with cardioprotective actions (statins, antihypertensive, and antiplatelet drugs) was noted when comparing patients with low to high-10-year ST1RE risk. The same result was observed in two studies conducted in Spain,^22^ the primary outcome of which was subclinical atherosclerosis. It is essential to highlight that no difference was noted concerning the use of cardioprotective drugs when comparing patients classified as having moderate (≥ 10 to < 20%) and high 10-year ST1RE risk, which possibly shows adhesion to guidelines published in 2014 that stated that patients with intermediate and high risk should receive similar preventive treatment.^40^ The level ≥10% has been used as the high 10-year risk in a model of a cardiovascular prediction risk in T1D by the Scottish Diabetes Research Network Epidemiology Group with external validation in the Swedish National Diabetes Register (NDR)^16^ and embraced by European Society of Cardiology.^14^ However, the NDR has suggested a level of ≥7.5% as high 5-year risk.^13^

In the present study, CVD occurred in 2.1% of patients, which was lower than that described in other countries such as USA, 3.5%^4^ and countries that took part in the EURODIAB, which ranged from ∼5 to 18%.^7^ Methodological differences regarding diagnostic criteria, socioeconomic, cultural, eating, lifestyle (including smoking) and the genetic background of the studied populations could account for this difference. We have observed a prevalence of CVD slightly, albeit significantly higher in men than in women. However, the association of CVD in patients with T1D regarding sex is still controversial.^4^^,^^5^^,^^7^^,^^10^ The current study’s findings are consistent with other studies regarding the main CVD, the IHD,^4^^,^^7^ and CVRFs: age, diabetes duration, inadequate glycemic, blood pressure, and LDL-cholesterol control. All these three traditional and modifiable CVRFs are still a challenging in routine clinical practice treating patients with T1D worldwide.^8^^,^^9^^,^^34^

In Brazil, as in many other countries, physicians see many patients in a limited time. Hence, identifying other CVRFs associated with 10-year moderate/high cardiovascular risk in the ST1RE engine might also help identify patients with such risk^.^ In this context, the presence of any type of DR, older age at diabetes diagnosis, higher levels of hs-CRP, and higher levels of uric acid were identified as factors associated with moderate/high 10-year cardiovascular risk according to the ST1RE engine. Notably, the presence of DR, mainly referable DR, has been included in different tools to stratify CV risk in patients with T1D.^14^^,^^16^^,^^17^ Moreover, higher levels of hs-CRP and uric acid have been associated with CVD and CKD.^32^^,^^33^ All the information mentioned above is essential for clinical decisions based mainly on observational studies due to the lack of randomized clinical controlled trials in patients with T1D.^34^

Several strengths and limitations should be addressed regarding this study. The main strength is that it comprised a large sample of patients that adequately represented the Brazilian population of patients with T1D, belonging to diverse multiethnic and socioeconomic backgrounds and coming from all the country’s geographic regions. Also, a uniform and standardized recruitment protocol was used regarding CVD risk assessment and the use of cardioprotective drugs. Finally, our statistical analysis showed some variables associated with moderate/high 10-year ST1RE risk that could help health practitioners working in very loaded settings to perform a screening in patients with an increased chance of a higher risk score and improve the use of the ST1RE engine in CVD prevention in routine clinical practice.

Nevertheless, our study has some limitations that must be mentioned. The first was the sample characteristics. All patients lived in large cities and received medical care in public health care centers from specialists; thus, patients who rely on primary care facilities and live in rural areas may not have been represented. However, the latter patients with T1D are the minority of those receiving treatment in Brazil. Secondly, our observational study’s cross-sectional design does not allow us to establish a causality association between the analyzed variables and 10-year ST1RE risk. However, recently the high 10-year ST1RE risk, was validate concerning CVD outcomes, but only in one Brazilian Diabetes center.^19^ Third, because this observational study used real-world clinical data, some urinary albumin information was missing, resulting in the exclusion of 193 patients. It is important to emphasize that missing data concerning urinary albumin is not unusual,^8^^,^^12^ and our sample presented a lower prevalence of missing data on this variable than other studies.^12^^,^^13^ Fourth, considering that our data have been collected from 2011‒2014 a contemporary new study with individuals with type 1 diabetes in Brazil is necessary .However it is important to highlight that several multicenter studies in type 1 diabetes continue to use their data for a long period of time, including the EURODIAB^7^ which demonstrates the difficulty in updating them. It is necessary for the National Health System in Brazil to establish a uniform database collection for all Diabetes Centers in the country, like the National Diabetes Register of Sweden to have frequently updated data.

In conclusion, the current study showed that most patients with T1D belonging to an ethnically highly admixed Latin American population presented with less than 10% risk of developing a cardiovascular event in the following ten years, according to the ST1RE risk engine. Moreover, clinical CVD was diagnosed mainly after over 15-years of T1D diagnosis. Higher age at diagnosis, the presence of DR, and higher levels of hs-CRP and uric acid might be considered possible predictors of moderate/high ST1RE risk. Lower use of cardioprotective drugs was noted in patients with moderate/ high 10-year ST1RE risk than recommended in 2014 ADA guidelines,^24^ which could translate into a gap in preventing CVD in Brazilian patients with T1D. Therefore, there is a need to establish strategies to improve cardiovascular and diabetes management risk in routine clinical practice since approximately 30% of our patients had moderate/high ST1RE risk.

## Abbreviations

T1D, Type 1 Diabetes; CVD, Cardiovascular Disease; CVRF, Cardiovascular Risk Factors; ST1RE, Steno Type 1 Risk Engine; ESC, European Society of Cardiology; QRISK3, Estimate cardiovascular Risk engine in United Kingdom; BrazDiab1SG, Brazilian Type 1 Diabetes Study Group; BNHCS, Brazilian National Health Care System; ADA, American Diabetes Association; ITR, Insulin Therapeutic Regimen; CSII, Continuous Subcutaneous Insulin Infusion; BMI, Body Mass Index; MS, Metabolic Syndrome; A1C, Glycated hemoglobin, HPLC, High Performance Liquid Chromatography; hs-CRP, high sensitivity-C-Reactive Protein; eGFR, estimated Glomerular Filtration Rate; DR, Diabetic Retinopathy; BIO, Binocular Indirect Ophthalmoscopy; NPDR, Non-Proliferative Diabetic Retinopathy; PDR, Proliferative Diabetic Retinopathy; FLI, Fatty Liver Index; ICD, International Code Disease; IBGE, Brazilian Institute of Geography and Statistics; IHD, Ischemic Heart Disease; SBP, Systolic Blood Pressure; ALT, Alanine Transaminase; AST, Aspartate Aminotransferase; NDR, Swedish National Diabetes Register.

## Ethics approval and informed consent to participate

Each local ethics committee has approved the study.

CEP/HUPE: 2769/2010.

CAAE: 0214.0.228.000-10.

## Informed consent for publication

Written informed consent was obtained from all participants prior to enrollment.

## Authors’ contributions

Marília Brito Gomes: Management and coordination responsibility for the research activity planning and execution; Preparation, creation and/or presentation of the published work, specifically writing the initial draft (including substantive translation).

Fernanda Oliveira Braga de Albuquerque: Preparation, creation and/or presentation of the published work, specifically writing the initial draft (including substantive translation).

Alessandra Matheus: Review and Editing; Conducting a research and investigation process, specifically performing the experiments, or data/evidence collection.

Luiz Henrique Canani: Review and Editing; Conducting a research and investigation process, specifically performing the experiments, or data/evidence collection.

Adriana Costa Forti: Review and Editing; Conducting a research and investigation process, specifically performing the experiments, or data/evidence collection.

Reine Marie Chaves: Review and Editing; Conducting a research and investigation process, specifically performing the experiments, or data/evidence collection.

Elizabeth João Pavin: Review and Editing; Conducting a research and investigation process, specifically performing the experiments, or data/evidence collection.

Renan Montenegro Junior: Review and Editing; Conducting a research and investigation process, specifically performing the experiments, or data/evidence collection.

Sergio A Dib: Review and Editing; Conducting a research and investigation process, specifically performing the experiments, or data/evidence collection.

João S Felicio: Review and Editing; Conducting a research and investigation process, specifically performing the experiments, or data/evidence collection.

Rosangela Roginski Réa: Review and Editing; Conducting a research and investigation process, specifically performing the experiments, or data/evidence collection.

Marcia Nery: Review and Editing; Conducting a research and investigation process, specifically performing the experiments, or data/evidence collection.

Carlos Antonio Negrato: Review and Editing; Conducting a research and investigation process, specifically performing the experiments, or data/evidence collection.

Luana Casari da Silva: Application of statistical, mathematical, computational, or other formal techniques to analyze or synthesize study data; Preparation, creation and/or presentation of the published work, specifically writing the initial draft (including substantive translation).

## Declaration of competing interest

On behalf of all authors, the corresponding author states that there is no conflict of interest.

## Data Availability

The datasets used and/or analyzed during the current study are available with the corresponding author upon reasonable request.
