# Supplementary material for: Cardiovascular risk estimation using the steno type 1 risk engine in Brazilian individuals with type 1 diabetes: a multicenter cross-sectional study
Source: Clinics (Sao Paulo). 2026 Sep 13;81:101082. doi: 10.1016/j.clinsp.2026.101082 (PMC13587914; doi:10.1016/j.clinsp.2026.101082)
Supplement: Supplementary file 1 [file mmc1.docx]

**CLINICS-D-25-00139_Supplementary Material**

**Supplementary Table 1** Sociodemographic data of the studied population.

| **Age, y** | 30.2 ± 11 |
| --- | --- |
| **Gender, F (%)** | 987 (56.1%) |
| **Diabetes duration, y** | 15.5 ± 9.3 |
| **Self-reported color-race, n (%)** |  |
| White | 958 (54.4%) |
| Years of school attendance, y | 12.2 ± 3.8 |
| **Economic status** |  |
| High | 53 (3%) |
| Medium | 801 (45.5%) |
| Low | 849 (48.2%) |
| Very low | 57 (3.2%) |
| **Geographic region, n (%)** |  |
| Southeast | 829 (47.1%) |
| South | 233 (13.2%) |
| North and Northeast | 490 (27.8%) |
| Midwest | 208 (11.8%) |
| **Level of care, n (%)** |  |
| Secondary | 651 (37%) |
| Tertiary | 1109 (63%) |
| **ITR, n (%)** |  |
| NPH or long-acting analogs | 86 (4.9%) |
| NPH or long-acting analogs plus regular or short-acting analogs | 1612 (91.6%) |
| CSII | 62 (3.5%) |
| **Physical activity** |  |
| Yes | 910 (51.7%) |
| No | 848 (48.2%) |
| **Smoking** |  |
| Yes | 227 (13.8%) |
| No | 1414 (86.2%) |

Data are presented as the means (SD) and n (%). y, year; F, Female; ITR, Insulin Therapeutic Regimen; CSII, Continuous Subcutaneous Insulin Injection.
